# Saving lives during the COVID-19 pandemic: the benefits of the first Swiss lockdown

**DOI:** 10.1186/s41937-021-00072-2

**Published:** 2021-08-12

**Authors:** Nicolò Gatti, Beatrice Retali

**Affiliations:** Institute of Economics (IdEP), Università della Svizzera Italiana, via G. Buffi 13, Lugano, CH-6900 Switzerland

**Keywords:** COVID-19, Lockdown, Saved lives, SIRDC model, SIR model, Behavioral responses, I18, D91, H12

## Abstract

The implementation of a lockdown to control the spread of the COVID-19 pandemic has led to a strong economic and political debate in several countries. This makes it crucial to shed light on the actual benefits of such kind of policy. To this purpose, we focus on the Swiss lockdown during the first wave of COVID-19 infections and estimate the number of potentially saved lives. To predict the number of deaths in the absence of any restrictive measure, we develop a novel age-structured SIRDC model which accounts for age-specific endogenous behavioral responses and for seasonal patterns in the spread of the virus. Including the additional fatalities which would have materialized because of the shortage of healthcare resources, our estimates suggest that the lockdown prevented more than 11,200 deaths between March and the beginning of September 2020.

## Introduction

Since the end of 2019, all countries in the world have experienced a rapid spread of the COVID-19 epidemic, which has required the fast development of appropriate policy responses to face the increasing number of infections, hospitalizations, and deaths. The majority of governments have therefore introduced different types of measures to reduce contacts among people. Such interventions have included bans on public events and gatherings of people and closures of national and regional borders, as well as school closures and the interruption of all non-essential business activities. These policies have been at the center of a heated debate, mainly due to their high economic and social costs.

A lockdown may have substantial negative effects on economic activities, leading to business disruption, job losses, and earnings reductions. Recent surveys reveal that at least 42% of young people experienced a deterioration of their career prospects and serious income losses (ILO, 2020). Such detrimental consequences in terms of learning outcomes and disposable income are also reverberated in lower levels of well-being and worse mental health conditions (OECD, 2020b; Cutler and Summers, 2020).

The aim of this paper is to evaluate the number of lives which a lockdown can potentially save. Given the economic costs implied by this policy, a reliable estimate of its benefits is crucial to understand whether its adoption is actually optimal (Gros, 2020). In order to address our research question, we focus on the lockdown implemented in Switzerland in response to the first wave of COVID-19 infections. To the best of our knowledge, the existing literature has not provided yet an estimate of the lives saved by the Swiss lockdown in spring 2020.

Taking advantage of a unique dataset about the universe of individuals who tested positive for the disease, we estimate the number of potentially saved lives by developing a novel SIRDC model, which allows to predict the daily amount of infections, hospitalizations and deaths for different age groups in the absence of lockdown. In particular, our model accounts for seasonal patterns characterizing the transmissibility of the virus (Atkeson, 2021) and includes age-specific endogenous behavioral responses (Cochrane, 2020). More specifically, we assume that not only individuals respond to changes in the death rate of their age group, but they are also *altruistic* and care about the well-being of other subjects. A basic SIR model, instead, would lead to overstate the impact of the policy right because it does not consider that citizens spontaneously reduce their contacts even in the absence of government interventions. To obtain a reliable estimate of saved lives, we also take into account potential *overflow* deaths due to hospital overcrowding. This is particularly relevant if we consider that the impossibility of providing proper hospital treatments, especially in intensive care units (ICU), results in a higher mortality risk also for younger subjects.

Our SIRDC model suggests that the absence of any policy intervention in Switzerland would have resulted into approximately 11,500 deaths by September 1, plus 1500 additional casualties due to the lack of available beds in intensive care units. Relying on a basic SIR model, instead, we would have predicted roughly 65,000 deaths, plus 62,000 fatalities due to the limited availability of healthcare resources. Such estimates would be in line with the simulations performed by the Imperial College COVID-19 Response Team. Neglecting hospital overcrowding, behavioral responses and seasonality, indeed, Flaxman et al. (2020) conclude that Switzerland would have reached 54,000 deaths by May 4. Our basic SIR model would deliver higher estimates only because we consider a time horizon which goes beyond May 4 and reaches the end of May, when the contagion fades out.

Our work is related to a growing literature concerning the impact of restrictive measures which limit the spread of an epidemic, especially after the outbreak of COVID-19. For instance, Zhang et al. (2020) show that contacts among people were reduced by more than seven times in China thanks to physical distancing policies, while Fang et al. (2020) document that the lockdown in Wuhan reduced the number of potential infections by almost 65%. Some studies have also attempted an evaluation of the monetary benefits associated to the lives saved by the lockdown (e.g., Greenstone and Nigam, 2020; Thunström et al., 2020). However, these analyses often rely on simulations based on early limited data (Verity et al., 2020).

This work contributes to the current literature about the COVID-19 pandemic from both a methodological and an empirical point of view. First, we develop a novel age-structured SIRDC model that accounts for seasonal patterns and age-specific endogenous behavioral responses, including both an egoistic and an altruistic component. Second, we provide an estimate of the severity of COVID-19 based on rich data concerning the entire period of the first wave of infections in Switzerland. Third, to the best of our knowledge, this is the first estimate of the number of lives saved by the first Swiss lockdown in spring 2020.

The rest of the paper is organized as follows. Section 2 introduces the Swiss context and the policies implemented during the first wave of the COVID-19 pandemic, between March and the beginning of September. Section 3 describes the data. Section 4 presents our model and the estimates of the potential number of deaths in the absence of containment measures. Section 5 focuses on overflow deaths due to hospital overcrowding. Section 6 concludes.

## Background

After the outbreak of the COVID-19 epidemic in China and in several European countries, at the end of February 2020 Switzerland started facing the spread of the virus, with an increasing number of infections. As a consequence, massive public health non-pharmaceutical interventions became the only viable strategy to limit the contagion.

Switzerland is a Confederation made up of 26 independent and sovereign cantons, so interventions can be planned and implemented both at national and cantonal levels. Indeed, some restrictive measures were already introduced, canceling several public events, on February 26 in the cantons at the border with Italy and France, where the first COVID-19 cases were reported^1^. Meanwhile, the first containment measure adopted at the national level by the federal government on February 28 was the banning of any event involving more than 1000 participants.

However, because of the rapidly increasing number of infections throughout the country, the Swiss federal government intervened with more stringent measures. In particular, on March 17, schools and non-essential economic activities were closed, while gatherings of more than five people were forbidden starting from March 20. Nevertheless, differently from other countries like Italy, Switzerland did not opt for a *strict* lockdown, with the general requirement to stay at home.

Although economic losses were expected to be severe also in a country with a high GDP per capita (World Bank, 2020) and *Human Development Index* score (United Nations, 2020), the the Federal Council (2020) aimed at avoiding an unsustainable burden in terms of infections and lost lives. Such concern was particularly reasonable considering that the Swiss population has increasingly aged over the last decades and more than 20% of people are older than 65, hence far more likely to develop serious illnesses or eventually die from COVID-19. In light of the constrained availability of healthcare facilities, moreover, it was necessary to prevent a scenario in which access to life-saving treatments would have been denied to patients in need.

After reaching a peak during the first half of April, the number of infections and, consequently, deaths started to exhibit a decreasing pattern. As a result, lockdown measures were progressively loosened. On April 27, several shops opened again, while schools restarted on May 11 and the activities in the majority of offices and facilities could take place again from June 8.

## Data

Our analysis is based on individual-level data released by the *Federal Office of Public Health* (FOPH) about the universe of individuals who tested positive for COVID-19 in Switzerland between February 24 and May 15, during the first wave of the epidemic^2^. For each positive case in a specific Swiss canton on a certain day, this dataset contains information about age and gender, as well as the date of the onset of the first symptoms. Furthermore, these data also report whether and when an individual was hospitalized, specifying if intensive care was required and providing the exact days on which the patient entered and left the intensive care unit. Finally, we know whether and when the person eventually died. Table 1 summarizes these data.

**Table 1:** Descriptive statistics by age group (by May 15)

	Age groups								
	0–9	10–19	20–29	30–39	40–49	50–64	65–79	80+	Total
**Panel A: positive cases**									
Number of cases	153	862	3801	4106	4768	8318	4393	4059	30460
Share of total cases	0.50%	2.83%	12.48%	13.48%	15.65%	27.31%	14.42%	13.33%	100%
Share of women	47.02%	58.58%	59.73%	57.20%	57.19%	50.29%	44.81%	60.70%	54.30%
**Panel B: hospitalizations and ICU**									
Hospitalizations	26	33	110	136	258	866	1275	1187	3891
Hospitalizations/cases	16.99%	3.83%	2.89%	3.31%	5.41%	10.41%	29.02%	29.24%	12.77%
ICU	1	1	5	15	27	132	239	78	498
ICU/cases	0.65%	0.12%	0.13%	0.36%	0.57%	1.59%	5.44%	1.92%	1.63%
Average days in ICU	–	–	–	4.33	10.50	16.25	11.41	8.66	11.30
**Panel C: deaths**									
Number of deaths	0	0	0	5	4	71	403	1112	1,595
Deaths/cases	0.00%	0.00%	0.00%	0.12%	0.08%	0.85%	9.17%	27.39%	5.24%
Share of women	0.00%	0.00%	0.00%	40.00%	25.00%	25.35%	31.27%	47.48%	42.32%

*Note:* This table summarizes the individual-level data released by the Federal Office of Public Health, which cover the period between February 24 and May 15, 2020. Panel A displays the number of officially reported positive cases, as well as the share of total cases in each age group and the share of women. Panel B shows the number of patients requiring hospitalization or intensive care in each age group, also expressed as a share of the total number of cases in the corresponding age group. In case of access to intensive care units, the data even report the exact dates of entry and exit, allowing to compute the average length of stay. Finally, panel C displays the number of COVID-related deaths in each age group, indicating the corresponding case fatality rate and the share of total fatalities occurred among women

In spite of relevant testing efforts, however, during the first wave of the pandemic, asymptomatic cases were largely undetected. Because of the limited availability of resources, only people with severe symptoms were tested. This is the reason why we derive information about seroprevalence from the study conducted in Geneva by Stringhini et al. (2020). In this way, it is possible to understand the extent to which younger subjects, who tend to be under-represented in the official data, were actually affected by the spread of the disease.

These data are complemented by the yearly cantonal statistics provided by the *Federal Statistical Office* about the resident population and the weekly number of deaths by age. As we will discuss in Section 4, we also exploit the Value of Statistical Life (VSL) to model the age-specific individual behavioral responses. The average VSL for the Swiss population is derived from the estimates released by the Federal Office for Spatial Development (2019)^3^. To obtain an age-specific VSL^4^, we rescale the estimates obtained by Murphy and Topel (2006) in the USA by means of the Swiss average value.

As far as the healthcare supply in Switzerland is concerned, we rely on several sources. The *Organization for Economic Cooperation and Development* (OECD) provides indicators about the number of total and acute care hospital beds per 1000 inhabitants, and the latest statistics available for Switzerland are for year 2018 (OECD, 2020a). We also refer to Rhodes et al. (2012), who estimated the number of intensive care beds in several European countries including Switzerland, expressing them as a percentage of total acute care beds. Besides, we rely on the information released by the *Swiss Society of Intensive Care Medicine* about the percentage of healthcare resources which could be exclusively allocated to COVID-19 patients. In order to derive the number of daily available beds, finally, we need statistics about the average length of stay in hospital and intensive care for COVID-19 patients. To this purpose, we exploit the FOPH dataset to compute the average number of days spent in ICU by these patients. In the case of individuals who were hospitalized but did not enter ICU, instead, FOPH data provide only the day of entrance, so we take advantage of the statistics available in Pellaud et al. (2020) about hospitalizations related to COVID-19 in Fribourg.

To estimate the number of overflow deaths due to hospital overcrowding, we finally need information about the mortality rates associated with being admitted to or rejected from hospital or ICU. While FOPH data allow to compute mortality rates for COVID-19 patients who received appropriate care, the corresponding estimates for rejected individuals will be taken from the literature (Greenstone and Nigam, 2020; Rojas, 2020), since Switzerland never faced the problem of overcrowded hospitals during the first wave of the pandemic.

## An estimate of potential *direct* deaths

The present section describes our estimates of the potential number of avoided *direct* deaths thanks to containment measures in Switzerland. The term “direct” refers to the fact that these estimates do not include the additional potential deaths due to hospital overcrowding, which will be computed in the next section. We now proceed with the following steps. First, we focus on the initial phase of the epidemic, when the growth of infections was not influenced yet by any restriction, to determine the parameters which allow to predict the subsequent spread of the contagion in a counterfactual scenario without mitigation policies. Second, we develop a novel SIRDC model to estimate the potential number of infections and the corresponding deaths between March and the beginning of September. To this purpose, we use an age-specific *imputed* infection fatality rate derived from the data^5^.

However, before proceeding with our analysis, we need to address a preliminary issue, which requires an adjustment of the data. Indeed, older people, who are more likely to exhibit severe symptoms, tend to be over-represented among positive cases, while younger (and often asymptomatic) individuals are systematically under-reported. Therefore, the total number of predicted infections in the *counterfactual* scenario cannot be attributed to the different age groups on the basis of the shares retrieved from the original data.

To circumvent this issue, we exploit the results obtained by Stringhini et al. (2020) from the seroprevalence tests conducted in Geneva. They not only estimate the overall seroprevalence in the population in each of the 5 weeks between April 6 and May 9, but they also compute how the relative risk varies depending on age. After computing the average value of seroprevalence over the 5 weeks, using the number of observations in each week as a weight, we exploit the specific relative risks to obtain the shares of people belonging to different age groups who have been actually infected in Geneva.

At this point, for each age group, we compute the ratio between the actual share of infected people in Geneva and the corresponding share of infected individuals in our data. Such ratio represents an age group-specific factor *k*_*a*_ measuring the extent to which each age group in the canton of Geneva is under-represented in the data (see Table 2). Since testing criteria in Switzerland are defined centrally by the FOPH, it is reasonable to assume that the factor *k*_*a*_ computed for Geneva can be applied to all the other cantons. Hence, after multiplying the number of reported cases in each age group by the corresponding adjustment factor *k*_*a*_, the issue of over- or under-representation of different groups is overcome (Table 3).

**Table 2:** Adjustment factors

Age	Estimated	Adjustment
	seroprevalence	factor *k*_*a*_
0–9	0.02808	44.908633
10–19	0.07546	30.858332
20–49	0.08774	7.7625095
50–64	0.06931	5.0841335
65+	0.04387	3.0066347

*Note:* This table reports the values of seroprevalence in different age groups inferred from the results of Stringhini et al. (2020) and the coefficients which should be multiplied by the official number of reported positive cases to predict the actual number of infections

**Table 3:** Descriptive statistics by age group after adjusting the data (by May 15)

	Age groups								
	0–9	10–19	20–29	30–39	40–49	50–64	65–79	80+	Total
**Panel A: positive cases**									
Number of cases	6871	26507	29366	31733	36911	42203	13181	12189	198961
Share of total cases	3.45%	13.32%	14.76%	15.95%	18.55%	21.21%	6.63%	6.13%	100%
**Panel B: hospitalizations and ICU**									
Hospitalizations	26	33	110	136	258	866	1275	1187	3891
Hospitalizations/cases	0.38%	0.12%	0.37%	0.43%	0.70%	2.05%	9.67%	9.74%	1.96%
ICU	1	1	5	15	27	132	239	78	498
ICU/cases	0.01%	0.00%	0.02%	0.05%	0.07%	0.31%	1.81%	0.64%	0.25%
Average days in ICU	–	–	–	4.33	10.50	16.25	11.41	8.66	11.30
**Panel C: deaths**									
Number of deaths	0	0	0	5	4	71	403	1112	1,595
Deaths/cases	0.00%	0.00%	0.00%	0.02%	0.01%	0.17%	3.06%	9.12%	0.80%
Share of women	0.00%	0.00%	0.00%	40.00%	25.00%	25.35%	31.27%	47.48%	42.32%

*Note:* This table summarizes the dataset which combines the individual-level data released by the Federal Office of Public Health (February 25–May 15, 2020) and the seroprevalence results inferred from Stringhini et al. (2020). Panel A displays the number of estimated positive cases, as well as the share of total cases attributed to each age group. Panel B shows the number of patients requiring hospitalization or intensive care in each age group, also expressed as a share of the total number of cases in the corresponding age group. In case of access to intensive care units, the data even report the exact dates of entry and exit, allowing to compute the average length of stay. Finally, panel C displays the number of COVID-related deaths in each age group, indicating the corresponding *imputed* infection fatality rate and the share of total fatalities occurred among women

### Estimating *R*_0_ during the early stage of the epidemic

As a first step, we estimate the *basic reproduction number* (*R*_0_) of the disease, which reveals the number of individuals who are infected by a single positive person during the initial phase of the epidemic^6^, when the population consists almost exclusively of susceptible individuals and the cumulative number of cases grows exponentially until some containment measures are introduced (Muggeo et al., 2020; Daddi and Giavalisco, 2020; Massad et al., 2005).

**Fig. 1:**
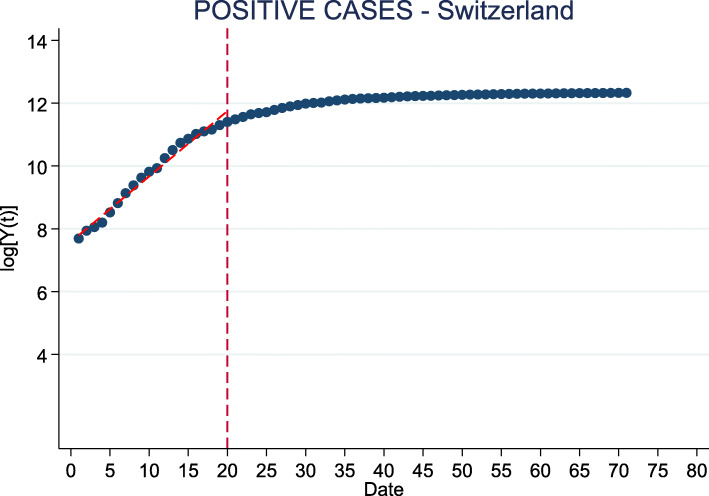
(Log) number of cumulative positive cases. *Note:* This figure shows the evolution over time of the logarithm of the cumulative number of infections after March 5. The number of cases represented here is the one obtained after adjusting the official number of reported cases in light of the seroprevalence estimates by Stringhini et al. (2020). The change in the slope which occurs around the 20th day reflects the end of an exponential growth of cases thanks to the implementation of restrictive measures in the country

The starting date of the epidemic is identified as the first day when an incidence of at least 20 cases of COVID-19 per 100,000 people is registered after the adjustment described above. The duration of the initial phase, before the materialization of any effect due to containment measures, is computed by estimating when the linear growth of the *logarithm* of the cumulative number of infections changes slope. In practice, we estimate a *hockey stick* regression model that allows to identify the *breakpoint* date at which the slope of this linear relationship changes^7^, as also displayed in Fig. 1:

1 $$ log \hspace*{0.05cm}(\mathbb{E}[Y_{t}]) = \beta_{0} + \beta_{1}t  $$

where *Y*_*t*_ is the cumulative number of infections at day *t*=1,2,...,*n*, after we have normalized the first day of the epidemic as day 1.

Table 4 reports the breakpoint dates estimated both for Switzerland and its seven macro-regions. Since the federal lockdown was announced on March 16, its effects are expected to be observed at most 10 days later, considering that the incubation period for COVID-19 amounts to 5 days and other 4.5 days pass on average between the onset of the first symptoms and the test. This timing is exactly reflected in our estimates, with an anticipated effect in French cantons and in Ticino, where some restrictions were introduced earlier.

**Table 4:** Estimates of *R*_0_ during the early phase of the epidemic

Region	Starting date	Breakpoint date	R_0_	*β*
Lake Geneva	6 March	23 March	2.2939	0.3955
Espace Mittelland	6 March	26 March	1.9005	0.3277
Northwestern Switzerland	5 March	25 March	1.9528	0.3367
Zurich	8 March	24 March	2.1808	0.3760
Eastern Switzerland	7 March	24 March	2.0553	0.3544
Central Switzerland	5 March	25 March	1.8601	0.3207
Ticino	3 March	22 March	2.1577	0.3720
**Switzerland**	5 March	24 March	2.0859	0.3596

*Note:* This table reports the estimated length of the early phase of the epidemic—characterized by an exponential growth of cases—and the corresponding *basic reproduction number* *R*_0_ in the main Swiss regions. The starting date is conventionally fixed when an incidence of at least 20 cases per 100,000 individuals is reached. The breakpoint date corresponds to a change in the growth rate of the cumulative number of cases due to containment measures (see Fig. 1). The value of *β* is retrieved by multiplying *R*_0_ and *γ*

In light of these results, it is finally possible to compute the value of *R*_0_ using the following equation (Massad et al., 2005; Daddi and Giavalisco, 2020):

2 $$ Y_{b} = Y_{1} * e^{(R_{0}-1) \gamma t}  $$

Here, *Y*_*b*_ is the cumulative number of infections on the breakpoint date, *Y*_1_ is the cumulative number of infections on the first day, while *γ* represents the *resolving* rate, so that $\frac {1}{\gamma }$ is the average infectious period during which an individual can transmit the virus to others. Such period can be expected to be similar to the incubation period and, indeed, according to Almeshal et al. (2020), it amounts to 5.8 days. Exploiting this value, we derive the estimates of *R*_0_ reported in Table 4. Given that the basic reproduction number *R*_0_ is defined as the product between the contact rate *β* and the average infectious period 1/*γ*, we can finally retrieve the value of *β*, which captures how the infection is transferred.

Table 4 reveals the existence of remarkable differences across Swiss regions in the intensity of the spread of the epidemic, which can also be explained by cultural heterogeneity (Mazzonna, 2020). A separate analysis of regions, however, would not allow to take into account the possibility that the contagion also spreads from one region to another, an aspect of key importance in a country where the degree of mobility is extremely high. Hence, in order to avoid underestimating the potential effects of lockdown measures, in the following sections of the paper we will rely on the number of infections, hospitalizations and deaths estimated at the national level.

### Imputed infection fatality rates

The most widely used measure for the severity of a disease is the infection fatality rate (IFR), which indicates the proportion of deaths among all infected individuals, including those who are asymptomatic or undiagnosed. After adjusting the data in light of seroprevalence results, we can actually estimate the whole number of cases in each age group. Hence, by taking the ratio between the number of reported deaths and the number of cases within each age group, we obtain an age group-specific *imputed* infection fatality rate *IFR*_*a*_ for COVID-19^8^. These estimates will now be exploited to fit our model and derive the potential number of *direct* deaths in the absence of restrictive measures.

### Direct deaths in the absence of restrictions

#### An age-structured SIRDC model with endogenous behaviors

The values of *R*_0_ and *β* determined above can be now exploited to fit a model which allows to simulate the spread of the COVID-19 epidemic in Switzerland in the absence of any mitigation policy. In particular, our aim is to improve the estimates which could be derived from a basic SIR model (see Appendix 1) by considering a more realistic counterfactual scenario in which people tend to reduce spontaneously their contacts also in the absence of any government intervention. Furthermore, following Atkeson (2021), we are also including in the model an additional component which accounts for seasonal variation in the spread of the virus. Indeed, as documented by the epidemiological literature (e.g., Park et al., 2020), the transmissibility of the virus changes during the year, reaching a peak towards the end of January.

As far as the time horizon of our predictions is concerned, we focus on the 180 days between March 5 and September 1. Indeed, the present analysis is meant to estimate the benefits associated to the lockdown implemented in response to the first wave of infections. Moreover, such focus allows us to avoid a potential bias in our estimates arising from factors which changed after summer and led to the insurgence of the second wave of infections. However, Appendix 4 also reports the results of our model when the time horizon is not restricted and we consider the entire period over which infections and deaths would occur.

We start from a simple SIRDC model (Villaverde and Jones, 2020), in which individuals can be in one of five possible states: Susceptible (S), Infectious (I), Resolving (R), Dead (D), and reCovered (C). Since we are interested in estimating how the number of potential infections and deaths varies with age, we distinguish eight age groups^9^.

Excluding vital dynamics (i.e., neglecting births and deaths that are unrelated to the epidemic, see Rowthorn and Maciejowski, 2020) and taking into account that the contagion may spread also across age groups, the model is described by the following system of five ordinary differential equations:

3 $$\begin{array}{*{20}l} \frac{dS_{a}}{dt} &= -\frac{\beta_{0} \sum_{a=1}^{8} I_{a} }{\sum_{a=1}^{8}N_{a}} * S_{a} \end{array} $$

4 $$\begin{array}{*{20}l} \frac{dI_{a}}{dt} &= \frac{\beta_{0} \sum_{a=1}^{8} I_{a} }{\sum_{a=1}^{8}N_{a}} * S_{a} - \gamma I_{a} \end{array} $$

5 $$\begin{array}{*{20}l} \frac{dR_{a}}{dt} &= \gamma I_{a} - \theta R_{a} \end{array} $$

6 $$\begin{array}{*{20}l} \frac{dD_{a}}{dt} &= \delta_{a} \theta R_{a}  \end{array} $$

7 $$\begin{array}{*{20}l} \frac{dC_{a}}{dt} &= (1 - \delta_{a}) \theta R_{a} \end{array} $$

with *a* indicating one of the eight age groups, *a*∈{1,...,8}. *N*_*a*_ represents the total population belonging to a given age group, while *N* represents the total population, which does not vary over time since vital dynamics are here neglected.

The number of subjects in each compartment varies over time, but the stock across the five states remains constant:

$$\begin{array}{*{20}l} &\sum_{a = 1}^{8}S_{a}(t) + \sum_{a = 1}^{8}I_{a}(t) +\sum_{a = 1}^{8}R_{a}(t) +\sum_{a = 1}^{8}D_{a}(t) \\ &+ \sum_{a = 1}^{8}C_{a}(t) = \sum_{a = 1}^{8}N_{a}(t) = N(t) = N \end{array} $$

The rate at which susceptible individuals in each age cohort *a* become infectious is $\frac {\beta _{0} \sum _{a=1}^{8} I_{a} }{\sum _{a=1}^{8}N_{a}} * S_{a} = \frac {\beta _{0} I S}{N}$. Hence, it depends on the share of infectious subjects in the total population, on the value of the contact rate *β*_0_, which mirrors the speed of the transmission of the disease, and on the amount of individuals who are still susceptible. Infectiousness resolves at rate *γ*. Once individuals are no longer in the state in which they can infect others, they move to the resolving state. In each period *t*, then, a constant fraction of individuals (*θ*) in every considered age group leaves the resolving compartment, ending in one of the two final stages: either dead (with probability *δ*_*a*_) or recovered (with probability (1−*δ*_*a*_))^10^. These last two states are permanent, that is, once in them, people can no longer change compartment. We set *β*_0_=0.3596 and *γ*=0.1724, while *δ*_*a*_ indicates age-specific mortality rates^11^. Finally, we set *θ*=0.1. This value reflects the $\frac {1}{\theta } = 10$ days which on average an individual spends with the disease before it resolves.

The system of differential equations can be recursively estimated to predict the daily number of people in each compartment. Since the analysis is performed at the national level, the initial conditions are represented by the individuals in each age group and compartment on March 5 (see Appendix 2). More in detail, the initial number of susceptible people in each age group is the number of individuals who had not been infected by March 5. Since the infectious period 1/*γ* is assumed to be 5.8 days on average, the initial number of infectious individuals is represented by the number of new infections occurred during the 5.8 days before March 5^12^. The initial number of people in the resolving state is given by all the subjects who were infected previously^13^. Only one person aged 72 had officially died from COVID-19 before March 5, while no subjects had recovered yet on this date. Finally, dividing these values by the total population, we obtain the shares of individuals who initially belong to each age group and compartment^14^.

At this point, following Cochrane (2020), we introduce in this framework an endogenous behavioral response common to all age groups. In other words, we suppose that when individuals start getting infected and dying, the contact rate *β* becomes lower, as people try to avoid the disease. Hence, we model the behavioral response as a function of the current death rate, according to the following equation:

8 $$ log(\beta_{t}) = log(\beta_{0}) - \alpha_{D} \frac{\Delta D_{t}}{N}  $$

where $D_{t} = \sum _{a = 1}^{8} D_{a, t}$ and $N = \sum _{a = 1}^{8} N_{a}$.

We calibrate *α*_*D*_ as in Cochrane (2020). Using Eq. 8, we assign values to *β*_0_,*β*_*t*_ and *ΔD*_*t*_ to obtain the parameter *α*_*D*_, which measures people’s sensitivity to changes in the death rates. *β*_0_ is the baseline contact rate (*β*_0_=0.3596), while *β*_*t*_ is the lowest value of *β* which is observed. Thus, the calculations based on our data reveal that *β*_*t*_=0.173^15^. The peak in the variation of the daily number of deaths in Switzerland is 25 deaths, so *ΔD*_*t*_=25. Finally, *N* is the total Swiss population in 2020. We recover *α*_*D*_=108,697.16.

However, we know that there is striking heterogeneity in mortality rates across age groups. If people’s behavior is affected by their perceived personal risk, behavioral responses could greatly vary by age and imposing a common differential equation for *β* could be an unrealistic assumption. Thus, we adapt the behavioral differential equation to introduce age-specific responses. We model the behavioral response of each age group as a function of both the death rate for that particular age group and a fraction of the death rates registered for the other age groups (introducing both an *egoistic* and an *altruistic* component).

First of all, we assume that individuals care to the maximum possible level (=1) to the death rate of people belonging to their own age group, so we keep a one-to-one relationship between $\frac {d\beta _{a}}{dt} $ and $\frac {dD_{a}}{dt}$. Second, we assume that individuals are, at least partially, altruistic, and adjust their behavior also in response to changes in the death rates of other age groups. However, they weight other people’s well-being less than their own, with an *altruism factor* equal to 0.27 (Long and Krause, 2017). Third, we assume that people do not give the same importance to the death rates of all the other age cohorts, but rather they adopt a societal perspective. In other words, individuals give more weight to the death rates of those age groups that have a higher VSL. Therefore, if we consider the perspective of age cohorts 0–9, 10–19, 30–39, 40–49, 50–64, 65–79, and 80+, and we normalize their VSL by giving value 1 to the highest VSL (i.e., that of the age group 20–29), we obtain the coefficients reported in column (1) of Table 5. When we adopt the perspective of individuals in group 20–29, we have slightly different normalized coefficients, since, excluding the VSL of that group, the highest VSL becomes that of the cohort aged 30–39. Normalizing it to 1, we obtain the coefficients displayed in column (2) of Table 5.

**Table 5:** Normalization coefficients by age group

	(1)	(2)
	Reference group:	Reference group:
Age	20–29	30–39
0–9	0.9126 = *ϕ*_1,3_	0.9302 = *ϕ*_1,4_
10–19	0.9514 = *ϕ*_2,3_	0.9697 = *ϕ*_2,4_
20–29	1 = *ϕ*_3,3_	–
30–39	0.9810 = *ϕ*_4,3_	1 = *ϕ*_4,4_
40–49	0.8557 = *ϕ*_5,3_	0.8723 = *ϕ*_5,4_
50–64	0.5936 = *ϕ*_6,3_	0.6050 = *ϕ*_6,4_
65–79	0.2729 = *ϕ*_7,3_	0.2781 = *ϕ*_7,4_
80+	0.0940 = *ϕ*_8,3_	0.0958 = *ϕ*_8,4_

*Note:* This table reports the normalized coefficients obtained by taking the ratio between the value of statistical life of each age group and the value of statistical life of a reference group. The reference categories are represented by the age groups 20–29 and 30–39, namely those characterized by, respectively, the first and second highest values of statistical life

Following Atkeson (2021), we finally include in Eq. 8 a parameter *ψ*(*t*) that captures seasonal patterns affecting the transmissibility of the virus:

9 $$ \psi(t) = \omega * (cos((t + \tau) * 2\pi / 365) - 1)/2  $$

where *ω* measures the amplitude of seasonal fluctuations and is set equal to 1, while *τ* identifies the peak in the transmission of the virus. In line with Atkeson (2021) and the epidemiological literature mentioned above (e.g., Park et al., 2020), we conventionally set this peak on January 31, thus *τ*=33^16^.

Putting everything together, we now have age-specific differential equations for the behavioral responses which can be included in the age-structured SIRDC model:

10 $$\begin{array}{*{20}l} &\frac{dS_{a}}{dt} = -\frac{\beta_{a} \sum_{a=1}^{8} I_{a}}{\sum_{a=1}^{8}N_{a}} * S_{a} \end{array} $$

11 $$\begin{array}{*{20}l} &\frac{dI_{a}}{dt} = \frac{\beta_{a} \sum_{a=1}^{8} I_{a} }{\sum_{a=1}^{8}N_{a}} * S_{a} - \gamma I_{a} \end{array} $$

12 $$\begin{array}{*{20}l} &\frac{dR_{a}}{dt} = \gamma I_{a} - \theta R_{a} \end{array} $$

13 $$\begin{array}{*{20}l} {}&\frac{dD_{a}}{dt} = \delta_{a} \theta R_{a} \end{array} $$

14 $$\begin{array}{*{20}l} {}&\frac{dC_{a}}{dt} = (1 - \delta_{a}) \theta R_{a} \end{array} $$

15 $$\begin{array}{*{20}l} {}&\frac{d\beta_{a}}{dt} =\frac{\beta_{0}}{exp\Big(\alpha_{D}\Big(\frac{dD_{a}}{dt} + 0.27 \Big(\sum\limits_{i=1, i\ne a}^{8} \phi_{i, 3} \frac{dD_{i}}{dt}\Big)\Big) - \psi\Big)} - \beta_{a} \\ {}&\hspace*{3.6cm}\text{for} \ a \in \{1, 2, 4, 5, 6, 7, 8\} \end{array} $$

16 $$\begin{array}{*{20}l} {}&\frac{d\beta_{a}}{dt} =\frac{\beta_{0}}{exp\Big(\alpha_{D}\Big(\frac{dD_{a}}{dt} + 0.27\Big(\sum\limits_{i=1, a\ne 3}^{8} \phi_{i, 4} \frac{dD_{i}}{dt}\Big)\Big) - \psi\Big)} - \beta_{a} \\ {}&\hspace*{5.3cm}\text{for} \ a \in \{3\} \end{array} $$

**Fig. 2:**
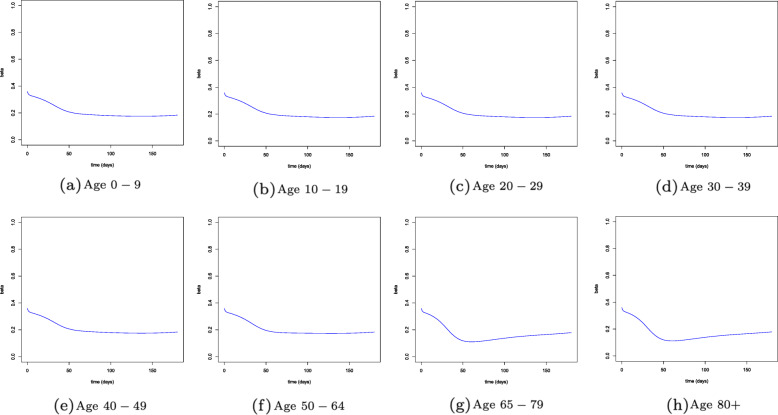
Contact rate by age group over time. *Note:* This figure displays the evolution over time of the contact rates of individuals belonging to different age groups. Such dynamics reflect the differences in the intensity of the behavioral responses of these subjects. In particular, older individuals—namely those who are more likely to suffer from the most severe consequences of the disease—tend to reduce their contact rates more substantially in response to an increase in the number of deaths

**Fig. 3:**
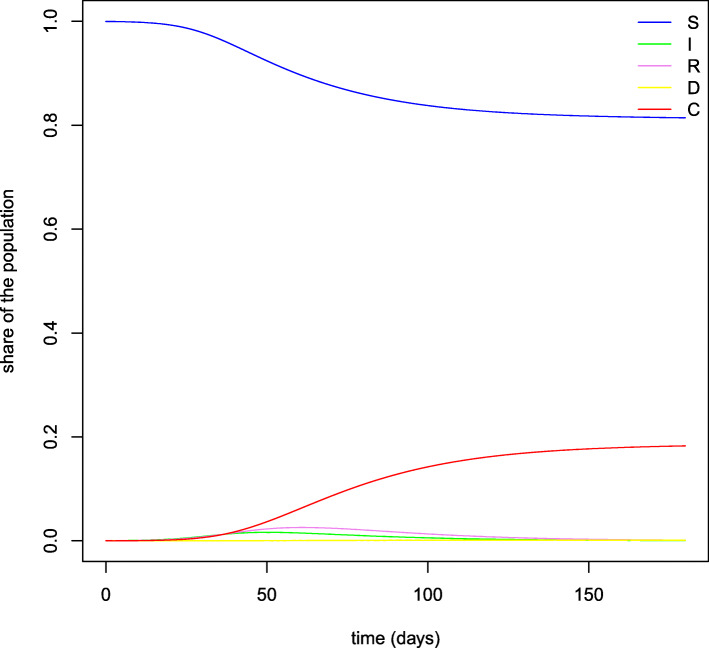
SIRDC model. *Note:* This figure plots the evolution of the daily shares of individuals in each compartment according to the predictions of our SIRDC model over the time period considered

These equations imply an immediate reduction of contact rates for older individuals, while younger people tend to reduce their interactions more slowly since the death rate for their age group is low or even null. Figure 2 shows the evolution over time of the contact rates by age cohort. As before, *N* is normalized to 1, so that *S*_*a*_,*I*_*a*_,*R*_*a*_,*D*_*a*_ and *C*_*a*_ represent the shares of population in each age group and compartment. As already mentioned, we consider a time horizon of 180 days. Therefore, instead of looking directly at the results for state *D*_*a*_ on September 1, direct deaths are obtained by applying the IFR to the cumulative number of infections predicted in each age group by that day. This allows to take into account the additional deaths which would have materialized in the first weeks of September. Figure 3 reports the evolution over time of the variables considered in our SIRDC model after aggregating the different age groups.

#### Results

Table 6 shows our estimates of the potential number of *direct* deaths^17^ in the absence of restrictive measures.

**Table 6:** Direct deaths (infections until September 1)

		SIR model			SIRDC model		
Age	Pop	Cases	*IFR*_*a*_	Deaths	Cases	*IFR*_*a*_	Deaths
0–9	871,211	712,403	0.0000%	0	172,233	0.0000%	0
10–19	844,092	690,167	0.0000%	0	166,857	0.0000%	0
20–29	1,045,160	854,592	0.0000%	0	206,098	0.0000%	0
30–39	1,228,988	1,004,847	0.0158%	159	242,444	0.0158%	38
40–49	1,198,240	979,793	0.0108%	106	236,544	0.0108%	26
50–64	1,810,157	1,480,214	0.1682%	2490	345,460	0.1682%	581
65–79	1,152,223	942,376	3.0574%	28,812	162,498	3.0574%	4968
80+	453,828	371,150	9.1148%	33,830	64,411	9.1148%	5871
Total	8,603,899	7,035,542		65,397	1,596,545		11,484

*Note:* This table reports the number of direct deaths predicted according to both a basic SIR model and our SIRDC model accounting for seasonality and endogenous behavioral responses. For each model, the table displays the estimated number of infections in each age group and the corresponding number of direct fatalities, as well as the *imputed* infection fatality rate used for the computation

According to our SIRDC model which accounts for citizens’ behavioral responses and for seasonal patterns, the spread of the virus in the absence of any government intervention would have caused almost 11,500 deaths within 6 months from the beginning of the pandemic (see also Table 14), especially among older age groups. Robustness evidence will be presented in Appendix 3, where we discuss an alternative approach to derive the infection fatality rates.

## An estimate of potential *overflow* deaths

The present section is dedicated to estimate the overflow deaths which would have occurred in a counterfactual scenario without lockdown measures. These fatalities would have resulted from hospitals reaching their capacity and being unable to serve some COVID-19 patients. In order to estimate them, we first need to quantify daily demand for specialized healthcare and daily supply of acute care and intensive care beds in Switzerland. Second, we need to assign mortality probabilities for cases requiring hospitalization or intensive care, both when appropriate care is provided or denied.

### Healthcare demand

The SIRDC model presented in Section 4.3.1 (as the basic SIR model in Appendix 1) allows us to compute the daily number of new cases within each age group. On each day *t*, the share of new cases in age group *a* can be computed as *NC*_*a*,*t*_=(*I*_*a*,*t*_−*I*_*a*,*t*−1_)+(*R*_*a*,*t*_−*R*_*a*,*t*−1_)+(*D*_*a*,*t*_−*D*_*a*,*t*−1_)+(*C*_*a*,*t*_−*C*_*a*,*t*−1_) in the SIRDC model. The actual number of cases is then obtained multiplying *NC*_*a*,*t*_ by the total Swiss population. In order to derive the demand for healthcare services by COVID-19 patients, we exploit our data to compute the share of infected individuals within each age group who were hospitalized or needed intensive care treatment^18^.

### Healthcare supply

As the survival probability of COVID-19 patients depends crucially on the provision of specialized care, we need precise information about the total number of available hospital and ICU beds in Switzerland. According to the OECD, in 2018, there were 3.6 acute care hospital beds per 1000 inhabitants in the country. Considering the population in 2020, the stock of curative hospital beds over the entire country turns out to be about 30,982 beds. According to Rhodes et al. (2012), then, 3.1*%* of these acute care beds are for intensive care, giving us a stock of 960 beds in Switzerland. This figure is in line with the estimate provided by the Swiss Society of Intensive Care Medicine, which sets the stock between 950 and 1000 beds in the 82 intensive care units present on the Swiss territory. For the moment, we do not consider the possibility to improve healthcare supply, although there is some evidence that the total stock of ICU beds could be increased by 50% (icumonitoring.ch, 2020).

However, healthcare resources cannot be allocated only to COVID-19 patients and, indeed, before the spread of the virus, the daily average occupation rate of hospital and ICU beds was, respectively, 74% and 75% (Federal Statistical Office, 2020; European Society of Intensive Care Medicin, 2020). We assume that 50% of the stock of acute care beds can be allocated to the treatment of COVID-19 patients and, following the Swiss Society of Intensive Care Medicine, we fix the available stock of ICU beds for individuals affected by COVID-19 at 56%.

The daily availability of beds depends also on the length of stay in hospital and intensive care for the average patient. Hence, we exploit the data released by the FOPH to calculate the average number of days spent by a COVID-19 patient in ICU, obtaining an estimate of 11.3 days. Some of the individuals admitted to the ICU spend some time before in acute care beds, for an average of 1.9 days. We notice in the data that when the patients pass through the hospitalization phase before receiving intensive care, the date of the test assessing whether they have contracted the virus or not is subsequent to the hospitalization date. We can speculate that these 120 people are first admitted to the hospital and then moved to ICU once confirmed to be positive for COVID-19.

With regard to patients who do not need ICU, instead, we cannot apply the same procedure described above to obtain the figure for hospitalizations, since we know when an individual enters the hospital, but the exit date is not available in the dataset. Therefore, we rely on Pellaud et al. (2020), who calculate several metrics in a retrospective cohort study about 196 hospitalized individuals with confirmed cases of COVID-19 in the Fribourg area. The average length of stay for COVID-19 patients who require hospitalization but not intensive care is 7 days.

Finally, daily supply is obtained dividing the stock of hospital and ICU beds which could be allocated to COVID-19 patients by the respective length of stay, obtaining an estimate of $0.5 * \frac {(30,982-960)}{7} = 2,144.39$ daily hospital beds and $0.56 * \frac {960}{11.3} = 47.58$ daily places available in ICU.

### Mortality rates

In order to estimate the number of *overflow* deaths, then, we need mortality rates for the cases in which people are admitted or not to the hospital or ICU. The individual data released by the FOPH also allow us to calculate the probability of death when patients receive appropriate care: indeed, the problem of overcrowding was never faced by Switzerland over the period covered by these data. In light of these data, the probability of dying for admitted patients is 17.9*%* in case of hospitalization and 52% in case of intensive care. These results are in line with those presented in international literature (Rojas, 2020; Greenstone and Nigam, 2020).

Since we cannot directly calculate the corresponding probabilities when the demand for healthcare cannot be accommodated, we follow Rojas (2020), who assumes that mortality increases threefold when a patient is rejected from a hospital (i.e., 53.7*%* in Switzerland). For ICU cases, we assume a survival probability of 10%, which is derived from the existing literature (Greenstone and Nigam, 2020; Ferguson et al., 2020). It is worth remarking that such assumptions imply that the mortality rates do not change depending on the age of the potential patient. This situation leads to a considerable number of overflow deaths also among younger people, explaining why these overflow deaths, compared to direct ones, are significantly higher in those categories. However, we do expect that reached a certain level of criticality, even younger people will face a significant risk of dying if left without proper healthcare interventions.

### Overflow deaths

Exploiting the daily demand and supply of hospital beds computed above, we can now predict the daily number of deaths due to the shortage of healthcare resources. More in detail, on days when *demand*≤*supply*, all people in need can receive appropriate care, and, therefore, survival probabilities are those estimated using FOPH data. When, instead, *demand*>*supply* and facilities reach their capacity (Greenstone and Nigam, 2020), for the individuals who do not receive healthcare, we apply the mortality probabilities of 53.7*%* and 90% for hospitalization and intensive care respectively.

Following the literature, we assume that age does not affect the probability of being rejected or admitted to healthcare facilities. In other words, the share of patients in each age group who do not receive appropriate care stays constant. For instance, if 20% of the cumulative number of patients cannot obtain a hospital bed on day *t*, that day 20% of patients belonging to each age group are assumed not to have received the needed care. We obtain the total number of overflow deaths over the considered time period by summing up across all days.

As reported in Table 7 (see also Table 15), our SIRDC model allows to predict slightly more than 1500 overflow deaths by September 1, all imputable to overcrowded ICUs. Such estimate is significantly lower in comparison to the one obtained by means of a basic SIR model. Endogenous individual responses and seasonal patterns, in fact, lead to a slower spread of the virus, flattening the number of new cases. As a result, since the fraction of new cases requiring hospitalization or intensive care remains constant, hospitals avoid reaching their maximum capacity.

**Table 7:** Overflow deaths (infections until September 1)

	SIR model			SIRDC model		
Age	Hospital	ICU	Total	Hospital	ICU	Total
0–9	772	256	1,028	0	19	19
10–19	140	57	197	0	4	4
20–29	799	194	993	0	14	14
30–39	967	669	1636	0	49	49
40–49	1549	889	2438	0	66	66
50–64	2856	4743	7599	0	336	336
65–79	16,083	17,888	33,971	0	858	858
80+	10,729	3680	14,409	0	178	178
Total	33,895	28,376	62,271	0	1524	1524

*Note:* This table reports the number of deaths due to the shortage of healthcare facilities predicted according to both a basic SIR model and our SIRDC model accounting for seasonality and endogenous behavioral responses. For each model, the table displays separately the number of overflow deaths which can be attributed to the lack of hospital (but not ICU) beds and to the lack of ICU beds

## Conclusions

The introduction of lockdown measures to limit the spread of the COVID-19 pandemic has been at the center of a heated economic and political debate in the majority of countries. Several studies have therefore attempted an evaluation of the benefits associated to such restrictive measures. Focusing on the lockdown implemented in Switzerland in March 2020, our paper contributes to this extensive literature from both a methodological and an empirical perspective.

In order to estimate the number of potentially saved lives during the first wave of the pandemic in Switzerland, we have developed a new SIRDC model which predicts the evolution of the epidemic in the absence of containment measures. In comparison to a basic model, our version includes additional features which make the counterfactual scenario more realistic. First of all, we incorporate age-specific endogenous behavioral responses. In other words, not only we consider that individuals would spontaneously reduce their contacts even in the absence of a government intervention, but we also account for the fact that this response varies depending on age. Furthermore, by including a seasonality component, we avoid to neglect that the transmissibility of the virus is not constant over time and, after reaching a peak in winter, tends to become very low in summer.

Our predictions about the daily number of infections, hospitalizations and deaths are based on rich individual-level data concerning COVID-19 cases in Switzerland. In particular, we exploit these data to derive the initial conditions and the necessary parameters to fit our model. We also predict the number of additional casualties which would have occurred because of the constrained availability of healthcare facilities. Although Switzerland did not face serious issues of hospital overcrowding during the first wave of the pandemic, in fact, the absence of containment measures would have led to a higher number of deaths because of the lack of hospital beds, especially in intensive care units.

Although the features of our SIRDC model allow to improve the reliability of predictions, results should always be interpreted cautiously. Indeed, they depend on the assumptions concerning the structure of model, the value of its parameters, and the utilization of healthcare resources.

According to our estimates, the absence of any policy intervention would have led to approximately 11,500 direct deaths within 6 months from the beginning of the pandemic, plus 1500 overflow fatalities related to hospital overcrowding. Considering the actual number of COVID-19-related deaths over the same time period, our results suggest that more than 11,200 lives were saved by the lockdown, as reported in Table 8. This is a largely relevant result, especially if we consider the relatively short time period under analysis (until September 1), which also includes the summer months during which the spread of the disease decreases spontaneously (see Appendix 4 for estimates covering a longer time horizon).

**Table 8:** Estimated number of saved lives (by September 1)

		SIR model			SIRDC model		
	Actual	Direct	Overflow	Excess	Direct	Overflow	Excess
0–9	1	0	1028	1027	0	19	18
10–19	0	0	197	197	0	4	4
20–29	0	0	993	993	0	14	14
30–39	5	159	1636	1790	38	49	82
40–49	6	106	2438	2538	26	66	86
50–64	90	2490	7599	9999	581	336	827
65–79	455	28,812	33,971	62,328	4968	858	5371
80+	1215	33,830	14,409	47,024	5871	178	4834
Total	1772	65,397	62,271	125,896	11,484	1524	11,236

*Note:* This table reports the number of saved lives in each age group according to both a basic SIR model and our SIRDC model accounting for seasonality and endogenous behavioral responses. The estimated number of saved lives is computed as the difference between the total number of predicted fatalities (direct and overflow) and the actual number of occurred deaths

## Appendix 1: An age-structured SIR model

The values of *R*_0_ and *β* determined in Section 4.1 can be exploited to fit a *susceptible-infected-recovered* (SIR) model which allows to simulate the evolution of the spread of the epidemic in Switzerland if containment measures had not been implemented. Since we are interested in estimating the number of potential infections which would have occurred in each age group, we build an age-structured SIR model following Deforche (2020), but letting the age groups be eight (i.e., 0–9; 10–19; 20–29; 30–39; 40–49; 50–64; 65–79; 80+) rather than only two.

According to this model, which allows contacts between all age groups *a*, at any time, each individual can be either Susceptible (S), Infectious (I), or Recovered (R). The last compartment not only includes those subjects who are not infectious any more, but also those who died because of the disease. Excluding vital dynamics (i.e., neglecting births and deaths that are unrelated to the epidemic, see Rowthorn and Maciejowski, 2020), the model is described by the following system of ordinary differential equations:

17 $$\begin{array}{*{20}l} &\frac{dS_{a}}{dt} = - \frac{\beta_{0} \sum_{a = 1}^{8} I_{a}}{\sum_{a = 1}^{8} N_{a}} * S_{a} & \end{array} $$

18 $$\begin{array}{*{20}l} &\frac{dI_{a}}{dt} = \frac{\beta_{0} \sum_{a = 1}^{8} I_{a}}{\sum_{a = 1}^{8} N_{a}} * S_{a} - \gamma I_{a} & \text{for} \ a\in \{1,..., 8\} \end{array} $$

19 $$\begin{array}{*{20}l} &\frac{dR_{a}}{dt} = \gamma I_{a} & \end{array} $$

The rate at which susceptible individuals in each age group *a* become infectious $\Big (\frac {\beta _{0} \sum _{a = 1}^{8} I_{a}}{\sum _{a = 1}^{8} N_{a}} * S_{a}\Big)$ depends on the share of infectious subjects in the total population, on the value of the contact rate *β*_0_, which mirrors the speed of the transmission of the disease, and on the remaining stock of susceptible individuals.

As previously mentioned, *γ* represents the rate at which infectiousness resolves: individuals who are no longer infectious move to the resolving state and cannot change compartment any more (Eksin et al., 2019; Toxvaerd, 2020). At each point in time, the cumulative stock of individuals across states remains constant: $\sum _{a = 1}^{8} (S_{a} + I_{a} + R_{a}) = \sum _{a = 1}^{8} N_{a} = N$, where *N* is the total population. Normalizing *N* to 1, *S*_*a*_,*I*_*a*_, and *R*_*a*_ are interpreted as the shares of the population belonging to each compartment.

At this point, the system of differential equations can be recursively estimated to predict the daily number of people in each compartment after the beginning of the epidemic. Since the analysis is performed at the national level, the initial conditions are represented by the individuals in each age group and compartment on March 5. We exploit the values of *β*_0_ and *γ* discussed before (*β*_0_=0.3596; *γ*=0.1724).

**Fig. 4:**
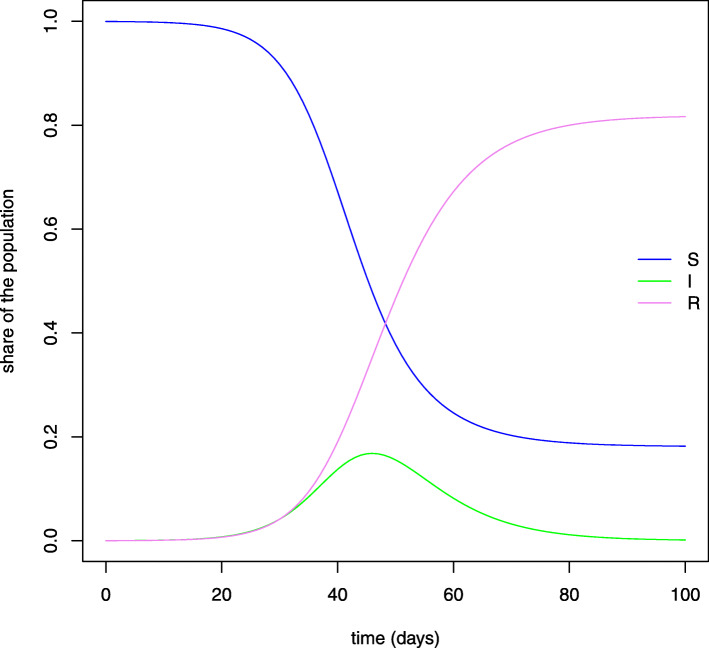
SIR model. *Note:* This figure plots the evolution of the daily shares of individuals in each compartment according to the predictions of a basic SIR model

Figure 4 plots the evolution over time of the predicted share of individuals who belong to each compartment when age groups are aggregated. It is interesting to observe that, when herd immunity is reached^19^, the epidemic continues to spread at a slower rate, since each person infects less than one other person. Thanks to this model, therefore, we can estimate the total number of infected people by the end of the pandemic, who correspond to the amount of people in state *R* when the number of susceptible individuals does not decrease any more and nobody else contracts the disease^20^. At this point, having predicted the total number of infections in each age group, the corresponding number of potential deaths can be derived through the infection fatality rate computed from the data.

## Appendix 2: SIR and SIRDC models—initial conditions

The initial values used to fit the SIR and SIRDC models are reported in Tables 9 and 10. The first subscript indicates the age group. These shares are calculated on March 5, 2020.

**Table 9:** Initial values—SIR model

Susceptibles	Infectious	Recovered
$S_{1,0} = \frac {871031}{8603899}$	$I_{1,0} = \frac {90}{8603899}$	$R_{1,0} = \frac {90}{8603899}$
$S_{2,0} = \frac {843844}{8603899}$	$ I_{2,0} = \frac {242}{8603899}$	$R_{2,0} = \frac {6}{8603899}$
$S_{3,0} = \frac {1044880}{8603899}$	$I_{3,0} = \frac {197}{8603899}$	$R_{3,0} = \frac {83}{8603899}$
$S_{4,0} = \frac {1228592}{8603899}$	$I_{4,0} = \frac {334}{8603899}$	$R_{4,0}= \frac {62}{8603899}$
$S_{5,0} = \frac {1197959}{8603899}$	$I_{5,0} = \frac {246}{8603899}$	$R_{5,0}= \frac {35}{8603899}$
$S_{6,0} = \frac {1809807}{8603899}$	$I_{6,0} = \frac {323}{8603899}$	$R_{6,0} = \frac {27}{8603899}$
$S_{7,0} = \frac {1152211}{8603899}$	$ I_{7,0} = \frac {69}{8603899}$	$R_{7,0} = \frac {12}{8603899}$
$S_{8,0} = \frac {453792}{8603899}$	$I_{8,0} = \frac {36}{8603899}$	$R_{8,0} = \frac {0}{8603899}$

*Note:* This table reports the shares of individuals in each compartment of the SIR model on March 5

**Table 10:** Initial values—SIRDC model

Susceptibles	Infectious	Resolving	Dead	Recovered
$S_{1,0} = \frac {871031}{8603899}$	$I_{1,0} = \frac {90}{8603899}$	$R_{1,0} = \frac {90}{8603899}$	$D_{1,0} = \frac {0}{8603899}$	$C_{1,0} = \frac {0}{8603899}$
$S_{2,0} = \frac {843844}{8603899}$	$I_{2,0} = \frac {242}{8603899}$	$R_{2,0} = \frac {6}{8603899}$	$D_{2,0} = \frac {0}{8603899}$	$C_{2,0} = \frac {0}{8603899}$
$S_{3,0} = \frac {1044880}{8603899}$	$I_{3,0} = \frac {197}{8603899}$	$R_{3,0} = \frac {83}{8603899}$	$D_{3,0} = \frac {0}{8603899}$	$C_{3,0} = \frac {0}{8603899}$
$S_{4,0} = \frac {1228592}{8603899}$	$I_{4,0} = \frac {334}{8603899}$	$ R_{4,0} = \frac {62}{8603899}$	$D_{4,0} = \frac {0}{8603899}$	$C_{4,0} = \frac {0}{8603899}$
$S_{5,0} = \frac {1197959}{8603899}$	$I_{5,0} = \frac {246}{8603899}$	$ R_{5,0} = \frac {35}{8603899}$	$D_{5,0} = \frac {0}{8603899}$	$C_{5,0} = \frac {0}{8603899}$
$S_{6,0} = \frac {1809807}{8603899}$	$ I_{6,0} = \frac {323}{8603899}$	$ R_{6,0} = \frac {27}{8603899}$	$D_{6,0} = \frac {0}{8603899}$	$C_{6,0} = \frac {0}{8603899}$
$S_{7,0} = \frac {1152211}{8603899}$	$ I_{7,0} = \frac {69}{8603899}$	$R_{7,0} = \frac {11}{8603899}$	$D_{7,0} = \frac {1}{8603899}$	$C_{7,0} = \frac {0}{8603899}$
$S_{8,0} = \frac {453792}{8603899}$	$I_{8,0} = \frac {36}{8603899}$	$R_{8,0} = \frac {0}{8603899}$	$D_{8,0} = \frac {0}{8603899}$	$C_{8,0} = \frac {0}{8603899}$

*Note:* This table reports the shares of individuals in each compartment of the SIRDC model on March 5

## Appendix 3: An alternative estimate of the infection fatality rate

Considering that several approaches have been proposed so far in the literature to estimate the infection fatality rate of COVID-19, we now back up the imputed IFR discussed in Section 4.2 by estimating the severity of the disease with an alternative methodology. More specifically, we follow the approach proposed by Rinaldi and Paradisi (2020), which relies on the use of administrative data concerning death counts and demographic information.

A potential concern regarding the *imputed* IFR reported in Table 6, indeed, is represented by the fact that official data about COVID-19 cases may misrepresent the actual number of deaths related to the spread of the virus. FOPH deaths data may present a downward bias because people might die at home (because of COVID-19) or in other non-medical facilities, and remain untested. This situation can be present if individuals decide not to go to the hospital, or they are not in a position to go. At the same time, official COVID-19 deaths data can present an upward bias since a fraction of those who died because of the pandemic were already severely ill individuals, who might have died over the following few weeks or months without the virus. Thus, COVID-19 has simply slightly anticipated their death.

In the attempt to correct for these biases, we use weekly administrative data about the deaths recorded between 2000 and 2020^21^ by the Federal Statistical Office, which also provides demographic information at the cantonal level^22^. We then elaborate these data to identify eight age groups (0–9; 10–19; 20–29; 30–39; 40–49; 50–64; 65–79; 80+) in the seven major Swiss regions (Lake Geneva, Espace Mittelland, North-West Switzerland, Zurich Region, Eastern Switzerland, Central Switzerland, and Ticino).

Exploiting such information, we build a Bayesian model which fits age-stratified mortality and demographic data for the seven regions between 2000 and 2020 over the weeks 11–19, namely those characterized by the COVID-19 outbreak. Specifically, starting from a simple standard binomial mortality mode, we assume that deaths are binomially distributed and in weeks affected by COVID-19 the baseline lethality rate is augmented by a factor that indicates the interaction between the IFR and the infection rate of COVID-19. Furthermore, we assume that mortality is not correlated between different age groups. The model is described with the following binomial equations:

20 $$\begin{array}{*{20}l} & D_{i,a,y} \sim \ \text{Binomial}(\delta_{a}, \ N_{i,a,y}) \hspace*{0.2cm} \text{for} \ y \in \{2000,..., 2019\} \end{array} $$

21 $$\begin{array}{*{20}l} & D_{i,a,2020} \sim \ \text{Binomial}(\delta_{a} + \delta_{a}^{Covid}*\theta_{i}, \ N_{i,a,2020}) \ & \end{array} $$

where *i* denotes the macro-region, *y* the year, and *a* one of the eight age groups (0–9; 10–19; 20–29; 30–39; 40–49; 50–64; 65–79; 80+). *D*_*i*,*a*,*y*_ and *N*_*i*,*a*,*y*_ are, respectively, the total deaths and population in macro-region *i*, year *y*, and age range *a*.

The baseline lethality rates *δ*_*a*_ are assumed to be constant across macro-regions and years, but can vary across age groups. Before 2020, the infection fatality rates $\delta _{a}^{Covid}$ are assumed to be equal to zero, while in 2020, they are heterogenous across age ranges and fixed in the other dimensions. Finally, the infection rates *θ*_*i*_ are region-specific but constant across age groups.

**Fig. 5:**
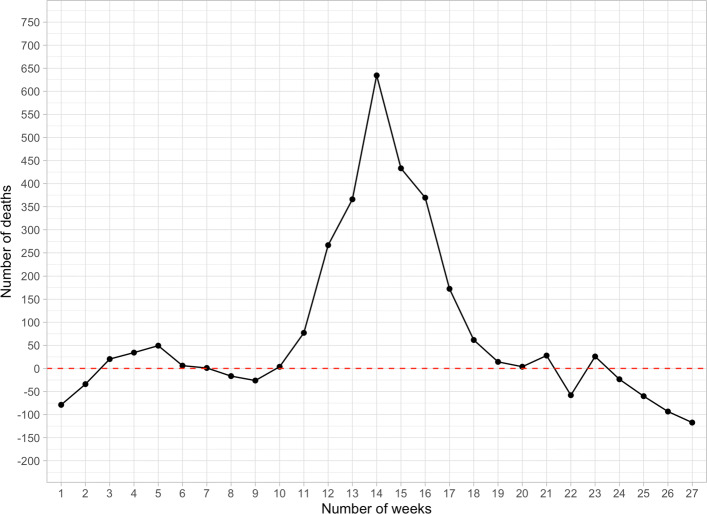
Excess mortality 2020 vs. mean 2000–2019. *Note:* This figure plots the weekly difference between the death counts in 2020 and the corresponding mean computed over the years between 2000 and 2019. During the first 10 weeks of 2020, excess mortality is approximately zero in expectation, while during the phase of the pandemic outbreak (weeks 11–19), excess mortality becomes significantly positive

The identifying assumption is that in the absence of the COVID-19 outbreak, the weekly deaths recorded in 2020 would have been the same on average as the ones in the previous 20 years. We provide visual evidence (Fig. 5) about the extent to which this assumption is satisfied. Indeed, over the first 10 weeks of 2020, excess mortality (calculated as the number of deaths in 2020 versus the average value of deaths over the years between 2000 and 2019) is substantially null. However, we cannot check whether the composition of the typologies of deaths changes over time and particularly in 2020, given that statistics on the causes of deaths are not available.

Using Markov Chain Monte Carlo procedures, we estimate an overall infection fatality rate for COVID-19 of 1.087123*%* (95% confidence interval 0.2899833%), with striking heterogeneity across age groups (see Table 11).

**Table 11:** Infection fatality rates by age group

Age groups	Median	Confidence interval
0–9	0.00016	(0.0000056–0.00110)
10–19	0.00023	(0.0000089–0.00130)
20–29	0.00014	(0.0000045–0.00094)
30–39	0.00019	(0.0000064–0.00120)
40–49	0.00023	(0.0000078–0.00150)
50–64	0.00023	(0.0000076–0.00160)
65–79	0.01300	(0.0031–0.03000)
80+	0.17000	(0.047–0.29000)

*Note:* This table reports the age group-specific infection fatality rates computed by means of the Bayesian approach, as well as the corresponding confidence intervals

As required with a Bayesian model, we specify priors for all the parameters we are interested in monitoring, i.e., $\delta _{a}, \delta _{a}^{Covid}, \theta _{i}$. We choose uninformative priors for all parameters:

22 $$\begin{array}{*{20}l} \delta_{a} \ & \sim \ \text{Uniform} [0, \ 0.1] \end{array} $$

23 $$\begin{array}{*{20}l} \delta_{a}^{Covid} \ & \sim \ \text{Uniform} [0, \ 0.3] \end{array} $$

24 $$\begin{array}{*{20}l} \theta_{i} \ & \sim \ \text{Uniform} [0, \ 0.2] \end{array} $$

To derive point estimates and respective 95% confidence intervals for the parameters of interest, we employ a Markov Chain Monte Carlo procedure that allows us to calculate the median and the confidence intervals of the posterior distributions of $\delta _{a}, \delta _{a}^{Covid}$, and *θ*_*i*_, using as model Eqs. (6) and (7)^23^. We draw 100,000 samples from the joint posterior distribution and use 50 independent chains. The burn in interval is fixed at 20,000, and the thinning interval is 30. Convergence is checked (and satisfied) visually with Gelman-Rubin diagnostic. Our estimates are robust to the definitions of alternative distributions of the priors.

Table 12 shows our estimates of the potential number of *direct* deaths in the absence of restrictive measures (both for SIR and SIRDC models), when we use the infection fatality rates estimated through this Bayesian approach. As previously mentioned, this approach leads to higher infection fatality rates, which result in more potential *direct* deaths, also among younger age groups.

**Table 12:** Direct deaths (infections until September 1)

		SIR model			SIRDC model		
Age	Pop	Cases	*IFR*_*a*_	Deaths	Cases	*IFR*_*a*_	Deaths
0–9	871,211	712,403	0.016%	114	159,980	0.016%	26
10–19	844,092	690,167	0.023%	159	154,907	0.023%	36
20–29	1,045,160	854,592	0.014%	120	191,341	0.014%	27
30–39	1,228,988	1,004,847	0.019%	191	225,456	0.019%	43
40–49	1,198,240	979,793	0.023%	225	219,719	0.023%	51
50–64	1,810,157	1,480,214	0.023%	340	331,345	0.023%	76
65–79	1,152,223	942,376	1.300%	12,251	181,062	1.300%	2354
80+	453,828	371,150	17.00%	63,095	51,367	17.00%	8732
Total	8,603,899	7,035,542		76,495	1,515,177		11,345

*Note:* This table reports the number of direct deaths predicted according to both a basic SIR model and our SIRDC model accounting for seasonality and endogenous behavioral responses. For each model, the table displays the estimated number of infections in each age group and the corresponding number of direct fatalities, as well as the *Bayesian* infection fatality rate used for the computation

It is worth underlining here that such differences in the infection fatality rates are also reverberated in the slight discrepancies between the number of cases predicted by the SIRDC model reported in Tables 6 and 12. According to our SIRDC model, indeed, individual behavioral responses depend on the number of daily deaths. Hence, changes in the fatality rate imply differences in the intensity of reduction of the contact rate *β*_*a*_ and in the number of predicted infections.

Since an alternative infection fatality rate leads to a different number of predicted infections, in Table 13, we report the corresponding overflow deaths due to the lack of available beds in intensive care units.

**Table 13:** Overflow deaths (infections until September 1)

	SIR model			SIRDC model		
Age	Hospital	ICU	Total	Hospital	ICU	Total
0–9	772	256	1,028	0	18	18
10–19	140	57	197	0	4	4
20–29	799	194	993	0	14	14
30–39	967	669	1636	0	47	47
40–49	1549	889	2438	0	63	63
50–64	2856	4743	7599	0	333	333
65–79	16,083	17,888	33,971	0	1032	1032
80+	10,729	3680	14,409	0	138	138
Total	33,895	28,376	62,271	0	1649	1649

*Note:* This table reports the number of overflow deaths due to the shortage of healthcare facilities predicted according to both a basic SIR model and our SIRDC model accounting for seasonality and endogenous behavioral responses. For each model, the table displays separately the number of overflow deaths which can be attributed to the lack of, respectively, hospital (but not ICU) and ICU beds

## Appendix 4: Results from the SIRDC model without restrictions on the time horizon

This Appendix reports the estimates derived from our SIRDC model accounting for seasonality and endogenous behavioral responses when we consider the entire time horizon until the contagion finally fades out and we do not restrict our attention only on the first 6 months after the beginning of the pandemic, before the outbreak of the second wave of infections.

**Fig. 6:**
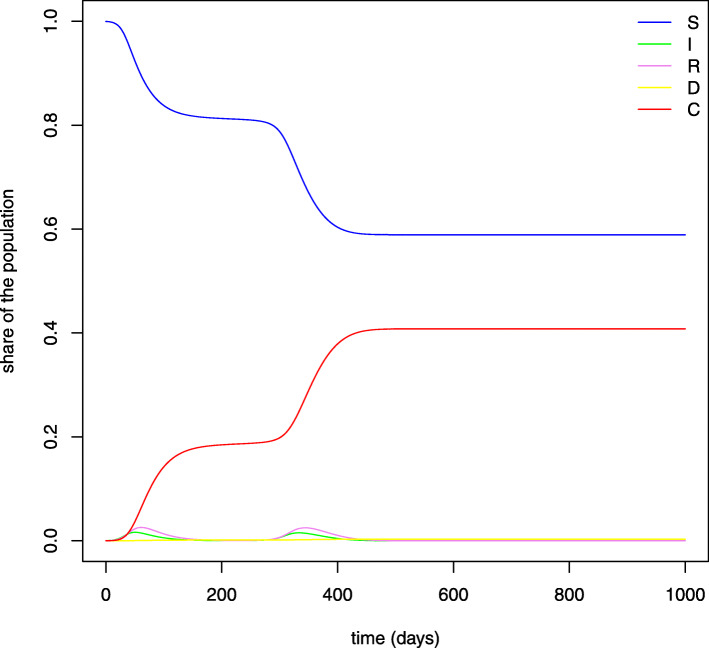
SIRDC model–time horizon: 1000 days. *Note:* This figure plots the evolution of the daily shares of individuals in each compartment according to the predictions of our SIRDC model in the absence of restrictions on the time horizon

Figure 6 shows that the model predicts also a second peak of infections after 200 days. The dynamics stabilizes after approximately 500 days, when 40% of Swiss individuals have been infected.

Tables 14 and 15 report the corresponding number of direct and overflow deaths by age group. In the absence of restrictions on the time horizon, our model would predict roughly 28,500 fatalities, more than twice the value over the first 6 months (see Tables 6 and 7).

**Table 14:** Direct deaths—SIRDC model

Age	Cases	*IFR*_*a*_	Deaths
0–9	381,119	0.0000%	0
10–19	369,223	0.0000%	0
20–29	456,055	0.0000%	0
30–39	536,458	0.0158%	85
40–49	523,409	0.0108%	57
50–64	764,014	0.1682%	1285
65–79	360,673	3.0574%	11,027
80+	142,946	9.1148%	13,029
Total	3,533,897		25,483

*Note:* This table reports the total number of direct deaths predicted by our SIRDC model accounting for seasonality and behavioral responses. The table displays the estimated number of infections in each age group and the corresponding number of direct fatalities, as well as the *imputed* infection fatality rate used for the computation

**Table 15:** Overflow deaths—SIRDC model

Age	Hospital	ICU	Total
0–9	0	40	40
10–19	0	9	9
20–29	0	30	30
30–39	0	104	104
40–49	0	138	138
50–64	0	703	703
65–79	0	1775	1775
80+	0	368	368
Total	0	3167	3167

*Note:* This table reports the total number of overflow deaths due to the shortage of healthcare facilities predicted by our SIRDC model accounting for seasonality and behavioral responses. The table displays separately the number of deaths which can be attributed to the lack of, respectively, hospital (but not ICU) and ICU beds

## Data Availability

The individual data used in this paper have been provided by the Federal Office of Public Health for the purpose of academic research. Such data are not publicly available to preserve patients’ anonymity, as the detailed personal information could potentially allow to identify specific subjects. The syntax used to analyze the data and derive the estimates is available from the authors upon request.

## Abbreviations

COVID-19: Coronavirus disease 2019
FOPH: Federal Office of Public Health
GDP: Gross Domestic Product
ICU: Intensive care unit
IFR: Infection fatality rate
OECD: Organization for Economic Cooperation and Development
SIR: Susceptible-Infectious-Recovered
SIRDC: Susceptible-Infectious-Resolving-Dead-reCovered
VSL: Value of Statistical Life

## Footnotes

1 The first official COVID-19 case in Switzerland was reported on February 25 in Ticino, the most southern canton at the border with Italy.

2 In addition, we exploit the number of deaths in each age group by the first week of September.

3 The most updated value refers to year 2017 and amounts to 6.7 million Swiss Francs. More information about the Swiss VSL is provided by Ecoplan (2016).

4 The VSL should exhibit a hump-shaped relationship with age (Aldy and Viscusi, 2008). Indeed, the VSL reflects not only life expectancy, but also other age-dependent characteristics such as education and career prospects. Hence, after increasing with age, the VSL starts declining when the individual turns approximately 30.

5 In order to check the robustness of our results, we also estimate the age-specific infection fatality rate of COVID-19 using an alternative approach based on a Bayesian model. See Appendix 3 for more details.

6 If *R*_0_=1, the number of infected people remains constant; if *R*_0_<1, the number of infected people decreases; if *R*_0_>1, the number of infected people increases.

7 If the cumulative number of infections grows exponentially during the early stage of the epidemic, the *log* of the cumulative number of infections exhibits a linear growth over time.

8 The value of *I**F**R*_*a*_ is null if no deaths are reported for age group *a*. The youngest individual who officially died from COVID-19 in Switzerland by May 15 is aged 31.

9 To implement this model, we have followed Deforche (2020), but identifying eight different age groups rather than only two. See Appendix 1 for more details.

10 Note that this dynamics collapses to that of a basic SIR model if we aggregate *R*_*a*_,*D*_*a*_, and *C*_*a*_.

11 Age-specific mortality rates are the imputed IFRs described in Section 4.2.

12 The initial number of infectious individuals on March 5 includes the infections registered between March 1 and March 5, plus 80% of the infections occurred on February 29.

13 Hence, individuals infected before February 28, plus 20% of those infected on February 29.

14 The adjustment based on seroprevalence results described before is meant to obtain reliable values at this stage of the analysis, avoiding an over-representation of older individuals.

15 We recovered the lowest observed value for *β* from *R*_0*t*_. Indeed, we first estimate the daily value for *R*_0*t*_, and we recover the corresponding *β*_*t*_ from the relationship $R_{0t} = \frac {\beta _{t}}{\gamma }$

16 Indeed, 33 days pass between January 31 and March 5.

17 Note that *I**F**R*_*a*_ is null for two of the three youngest age groups, as no deaths are reported in the official data. The youngest individual who has officially died because of COVID-19 in Switzerland is aged 31.

18 We distinguish between people needing a hospital bed, but not intensive care, and people needing intensive care.

19 Herd immunity is reached 46 days after March 5, i.e., on April 20.

20 Quite reassuringly, it is also possible to observe that the cumulated number of infections predicted by the model during the first days after March 5, when containment measures were not in place yet, are actually in line with those observed in the data.

21 Data provide information about gender, age group (5 years bin), and cantonal residence.

22 Data provide information on the total population, by gender and age.

23 The likelihood function is composed of 5 equations for each combination macro-region—age group, for a total of 21∗7∗8=1176 equations

## References

[CR1] Aldy J.E., Viscusi W.K. (2008). Adjusting the value of a statistical life for age and cohort effects. The Review of Economics and Statistics.

[CR2] Almeshal A.M., Almazrouee A.I., Alenizi M.R., Alhajeri S.N. (2020). Forecasting the spread of COVID-19 in Kuwait using compartmental and logistic regression models. Applied Sciences.

[CR3] Atkeson, A. (2021). A parsimonious behavioral SEIR model of the 2020 COVID epidemic in the United States and the United Kingdom. NBER Working Papers 28434, National Bureau of Economic Research. 10.3386/w28434.

[CR4] Cochrane, J.H. (2020). A SIR model with behavior. https://johnhcochrane.blogspot.com/2020/05/an-sir-model-with-behavior.html. Accessed 9 Mar 2021.

[CR5] Cutler D.M., Summers L.H. (2020). The COVID-19 pandemic and the *$*16 trillion virus. Jama.

[CR6] Daddi, E., & Giavalisco, M. (2020). Early forecasts of the evolution of the COVID-19 outbreaks and quantitative assessment of the effectiveness of countering measures. arXiv preprint arXiv:2004.08365.

[CR7] Deforche, K. (2020). An age-structured epidemiological model of the Belgian COVID-19 epidemic. *medRxiv*. 10.1101/2020.04.23.20077115.

[CR8] Ecoplan (2016). Empfehlungen zur Festlegung der Zahlungsbereitschaft für die Verminderung des Unfall und Gesundheitsrisikos (value of statistical life). *Forschung und Beratung in Wirtschaft und Politik*. Available at: https://www.are.admin.ch/are/de/home/suche.html#value%20of%20statistical%20life.

[CR9] Eksin C., Paarporn K., Weitz J.S. (2019). Systematic biases in disease forecasting–the role of behavior change. Epidemics.

[CR10] European Society of Intensive Care Medicin (2020). Coronavirus – public health emergency. https://www.esicm.org/resources/coronavirus-public-health-emergency. Accessed 10 Nov 2020.

[CR11] Fang H., Wang L., Yang Y. (2020). Human mobility restrictions and the spread of the novel coronavirus (2019-ncov) in China. Journal of Public Economics.

[CR12] Federal Council (2020). Bundesrat verschärft Massnahmen gegen das Coronavirus zum Schutz der Gesundheit und unterstützt betroffene Branchen. https://www.admin.ch/gov/de/start/dokumentation/medienmitteilungen/bundesrat.msg-id-78437.html. Accessed 17 May 2021.

[CR13] Federal Office for Spatial Development (2019). Value of Statistical Life (VOSL): Empfohlener Wert der Zahlungsbereitschaft für die Verminderung des Unfall und Gesundheitsrisikos in der Schweiz. *Bundesamt für Raumentwicklung*. Available at: https://www.are.admin.ch/are/de/home/suche.html#value%20of%20statistical%20life.

[CR14] Federal Statistical Office (2020). Medizinische Statistik der Krankenhäuser. https://www.bfs.admin.ch/bfs/de/home/statistiken/gesundheit/erhebungen/ms.html. Accessed 10 Nov 2020.

[CR15] Ferguson, N., *et al* (2020). Report 9: impact of non-pharmaceutical interventions (NPIs) to reduce COVID-19 mortality and healthcare demand. Imperial College COVID-19 Response Team, London, 16 March 2020. https://www.imperial.ac.uk/mrc-global-infectious-disease-analysis/covid-19/report-9-impact-of-npis-on-covid-19/.

[CR16] Fernández-Villaverde, J., & Jones, C.I. (2020). Estimating and simulating a SIRD model of COVID-19 for many countries, states and cities.. NBER Working Papers 27128. National Bureau of Economic Research. https://www.nber.org/system/files/working_papers/w27128/w27128.pdf. 10.1016/j.jedc.2022.104318 PMC8799324 35125563

[CR17] Flaxman, S., *et al* (2020). Estimating the effects of non-pharmaceutical interventions on COVID-19 in Europe. *Nature*, *584*(7820), 257–261. 10.1038/s41586-020-2405-7 32512579

[CR18] Greenstone, M., & Nigam, V. (2020). Does social distancing matter? Working Papers 2020-26, Becker Friedman Institute for Research in Economics. https://bfi.uchicago.edu/wp-content/uploads/BFI_WP_202026.pdf.

[CR19] Gros, D. (2020). The great lockdown: was it worth it?*CEPS Policy Insights*, *2020-11*. https://www.ceps.eu/ceps-publications/the-great-lockdown/.

[CR20] icumonitoring.ch (2020). Near-real time monitoring of intensive care occupancy. https://icumonitoring.ch/. Accessed 10 Nov 2020.

[CR21] ILO (2020). Youth and COVID-19: impacts on jobs, education, rights and mental well-being. Survey report 2020. *ILO Global Reports*. https://www.ilo.org/global/topics/youth-employment/publications/WCMS_753026/lang--en/index.htm.

[CR22] Long M.C., Krause E. (2017). Altruism by age and social proximity. PLoS ONE.

[CR23] Massad E., Burattini M.N., Lopez L.F., Coutinho F.A. (2005). Forecasting versus projection models in epidemiology: the case of the SARS epidemics. Medical Hypotheses.

[CR24] Mazzonna, F. (2020). *Cultural differences in COVID-19 spread and policy compliance: evidence from Switzerland. Covid Economics 33, 30 June 2020*, (pp. 163–185): CEPR Press.

[CR25] Muggeo, V., Sottile, G., Porcu, M. (2020). Modelling COVID-19 outbreak: segmented regression to assess lockdown effectiveness. Research Gate. 10.13140/RG.2.2.32798.28485.

[CR26] Murphy K.M., Topel R.H. (2006). The value of health and longevity. Journal of political Economy.

[CR27] OECD (2020a). Hospital beds. https://data.oecd.org/healtheqt/hospital-beds.htm. Accessed 17 Oct 2020.

[CR28] OECD (2020b). Youth and COVID-19: response, recovery and resilience. *OECD Policy Responses to Coronavirus*. https://www.oecd.org/coronavirus/policy-responses/youth-and-covid-19-response-recovery-and-resilience-c40e61c6/.

[CR29] Park S., Lee Y., Michelow I.C., Choe Y.J. (2020). Global seasonality of human coronaviruses: a systematic review. Open Forum Infectious Diseases.

[CR30] Pellaud, C., *et al* (2020). Characteristics, comorbidities, 30-day outcome and in-hospital mortality of patients hospitalised with COVID-19 in a Swiss area – a retrospective cohort study. *Swiss Medical Weekly*, *150*, w20314. 10.4414/smw.2020.20314. Accessed 09 Mar 2021. 10.4414/smw.2020.20314 32662869

[CR31] Rhodes A., Ferdinande P., Flaatten H., Guidet B., Metnitz P.G., Moreno R.P. (2012). The variability of critical care bed numbers in Europe. Intensive care medicine.

[CR32] Rinaldi, G., & Paradisi, M. (2020). An empirical estimate of the infection fatality rate of COVID-19 from the first Italian outbreak. *medRxiv*. 10.1101/2020.04.18.20070912.

[CR33] Rojas, I. (2020). On the economic benefits and costs of COVID-19 mitigation measures in Mexico. Available at SSRN: https://ssrn.com/abstract=3592209; 10.2139/ssrn.3592209.

[CR34] Rowthorn R., Maciejowski J. (2020). A cost–benefit analysis of the COVID-19 disease. Oxford Review of Economic Policy.

[CR35] Stringhini, S., *et al* (2020). Seroprevalence of anti-SARS-CoV-2 IgG antibodies in Geneva, Switzerland (SEROCoV-POP): a population-based study. *The Lancet*. 10.1016/S0140-6736(20)31304-0. 10.1016/S0140-6736(20)31304-0 PMC7289564 32534626

[CR36] Thunström L., Newbold S.C., Finnoff D., Ashworth M., Shogren J.F. (2020). The benefits and costs of using social distancing to flatten the curve for COVID-19. Journal of Benefit-Cost Analysis.

[CR37] Toxvaerd, F. (2020). Equilibrium social distancing. Cambridge Working Papers in Economics 2021, Faculty of Economics, University of Cambridge. 10.17863/CAM.52489.

[CR38] United Nations (2020). Human development indicators. http://hdr.undp.org/en/countries/profiles. Accessed 9 Mar 2021.

[CR39] Verity, R., *et al* (2020). Estimates of the severity of coronavirus disease 2019: a model-based analysis. *The Lancet Infectious Diseases*. 10.1016/S1473-3099(20)30243-7. 10.1016/S1473-3099(20)30243-7 PMC7158570 32240634

[CR40] World Bank (2020). World Bank Indicators - GDP per capita. https://data.worldbank.org/indicator/NY.GDP.PCAP.CD?locations=CH&most_recent_value_desc=true. Accessed 9 Mar 2021.

[CR41] Zhang, J., *et al* (2020). Changes in contact patterns shape the dynamics of the COVID-19 outbreak in China. *Science*, *368*(6498), 1481–1486. 10.1126/science.abb8001. 10.1126/science.abb8001 PMC7199529 32350060

